# The potential of corn-soybean intercropping to improve the soil health status and biomass production in cool climate boreal ecosystems

**DOI:** 10.1038/s41598-019-49558-3

**Published:** 2019-09-11

**Authors:** Muhammad Zaeem, Muhammad Nadeem, Thu Huong Pham, Waqar Ashiq, Waqas Ali, Syed Shah Mohioudin Gilani, Sathya Elavarthi, Vanessa Kavanagh, Mumtaz Cheema, Lakshman Galagedara, Raymond Thomas

**Affiliations:** 1 School of Science and the Environment, Memorial University of Newfoundland, Corner Brook, A2H 5G4 Canada; 2 Department of Environmental Sciences, COMSATS University of Islamabad, Vehari, 61100 Pakistan; 3 Department of Agriculture and Natural Resources Delaware State University, 1200N Dupont Hwy, Dover, DE 19901 USA; 4 Department of Fisheries, and Land Resources, Government of Newfoundland and Labrador, Pasadena, NL A0L 1K0 Canada

**Keywords:** Plant sciences, Environmental sciences

## Abstract

Intercropping (IC) is a promising approach used to improve soil health and sustainable crop production. However, it is unknown whether IC improve the soil health status and biomass productivity of crops cultivated in podzols under cool climate in boreal ecosystems. Two silage corn and three forage soybean genotypes were cultivated either as inter or monocrop (MC) treatments in a randomized complete block design. IC resulted in 28% increase in total forage production (FP). A reduction in rhizosphere soil pH (RS-pH) was observed in the IC treatments. Conversely, the rhizosphere soil acid phosphatase (RS-APase) activity was significantly higher (26–46%) in the IC treatments and occurred concomitant with a significant increase in available phosphorus (RS-P_available_) (26–74%) in the rhizosphere. Furthermore, IC enhanced the active microbial composition and strong positive correlations were observed between RS-P_available_, RS-APase, microbial biomass and FP; while RS-pH was negatively correlated with FP, RS-APase and RS-P_available_. These findings suggested silage corn intercropped with forage soybean could be a viable approach to enhance FP through improved active microbial community structure, RS-APase activity and RS-P_available_ when cultivated on podzols in cool climate boreal ecosystem.

## Introduction

The global population is anticipated to increase by 33% from 7.2 billion to 9.6 billion by 2050^1^. As such, the demand for sustainable food production systems to support this expanding population under different climate change and land use scenarios will be a challenge to food security^2^. This is of particular relevance in cool climatic regions where the current population are expanding, but there are limitations to increasing agriculture productions in these regions due to short growing season, limited agricultural crop varieties suitably adapted for production in these regions, low availability of arable land and poor soils (podzols)^3^. Therefore, there is a need to have innovative or improved cropping systems that will permit intensive, but sustainable crop production to provide food for livestock and the expanding population in cool climatic regions across the globe.

Intercropping (IC) is defined as the growing of two or more crops simultaneously on the same piece of land^4–6^, and is increasingly being adopted as a more sustainable practice in modern agricultural production systems throughout the world^7–9^. Conversely, monocropping [MC] (*growing of one crop on the same piece of land*) is less sustainable though the dominant production system in industrial agriculture. A common and popular index used to evaluate the yield advantage or agronomic performance of IC over MC is the land equivalent ratio (LER)^10–14^. The LER is defined as the relative land area required in MC to produce the same yield as in an IC production system. One advantage of LER is that it can be used to assess the agronomic performance, as well as determine the competitiveness for land resources between two crops or two cropping systems; for instance, MC systems versus IC systems^15^.

Cereal-legume IC is a crop production system utilized to improve productivity and sustainability under diverse environmental conditions. The improved productivity observed in this production system have been associated with increased levels of available phosphorus (P) in the root rhizosphere of IC species^16^, more stable yield, superior land resource utilization or conservation^17–20^, and enhanced pest or weed control^21–23^. Furthermore, cereal- legume IC can also enhance the phosphatase enzyme activity and available P in the soil due to rhizosphere acidification by the legumes used in the cropping system^24^.

The major form of P in agricultural soils is organic P, and can only be used after hydrolyzation by phosphatase enzymes^25–28^. Soil microbes are major sources of phosphatase and other soil enzymes, and their role in hydrolyzing or mineralizing organic P sources are well documented^7,26–29^. Furthermore, IC have been observed to improve the diversity and richness of the active soil microbial community resulting in superior mobilization of nutrients in the root rhizosphere^7,9,30^.

Phospholipid fatty acids (PLFAs) obtained from the membranes of soil microbes can be used as biomarkers to study or assess the active microbial community composition of gram-positive (G+) or gram-negative (G−) bacteria, actinomycetes, archaea, fungi (F) and protozoa living in the root rhizosphere^31–33^. Soil microbes are known to be very sensitive to minor changes in the soil environment, and thus have been extensively used to compare different crop management practices and land use systems^34^, as well as assess nutrient stresses^35,36^ in the root rhizosphere. Therefore, PLFA profiling is an efficient way to evaluate the living (active) microbial community in the soil, and can be used as a proxy to assess the soil health status and soil quality^37^. Previous studies have demonstrated that IC can modify the dominant microbial species composition, and structure in the root rhizosphere^12,31–33,38^.

Corn (*Zea mays* L.) is the third most important cereal crop cultivated globally^39^. It is an exhaustive crop that depletes the soil nutrients^40^. Soybean on the other hand, is a restorative crop that can replenish the soil with nutrients. Consequently, a number of studies have evaluated the effects of IC corn with peanut^41^, chickpea^26^, cowpea^42^, and faba bean^43^ on the soil chemical and biological properties. However, there is lack of information on the effects of silage corn intercropped with forage soybeans under cool climates in boreal ecosystems. In particular, there is no information available in the literature to the best of our knowledge on the effects of vine type forage soybeans intercropped with silage corn on the soil heath status and forage production following cultivation in podzolic soil under cool climatic conditions in boreal ecosystems. We hypothesized that silage corn intercropped with forage soybean could enhance not only forage yield, but also improve the soil health status during forage production in podzols present in cool climate boreal ecosystems. Therefore, the objectives of the current study were to evaluate: 1) the potential of silage corn and forage soybean genotypes IC to enhance forage production, 2) effects of IC on soil nutrient status and the active microbial communities, and 3) relationship between the active microbial community structure, soil health status and agronomic performance in cool climate boreal ecosystem.

## Results

### Plant performance indicators (Forage production, chlorophyll contents, and plant height)

**Figure 1:**
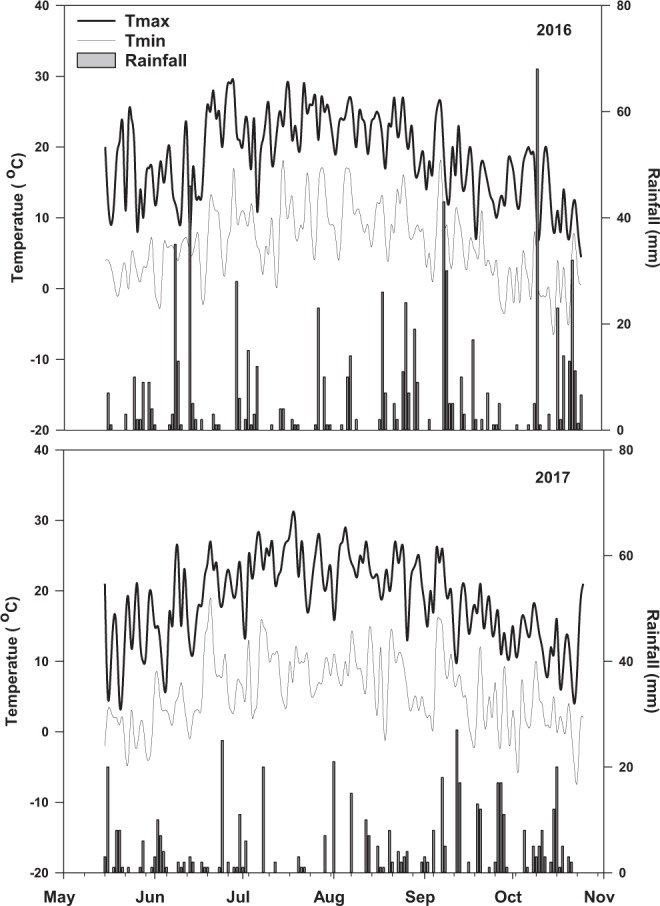
The average maximum (Tmax) and minimum (Tmin) temperature and total rainfall during 2016 and 2017 growing seasons.

Total eleven experimental treatments for two corn and three soybean genotypes is presented in Table 1. Out of total 37 observed PLFAs (Table 2), 27 were used to as biomarkers to identify the various microbial groups living under prevailing climatic conditions (Fig. 1). The forage production (FP), chlorophyll contents and plant height were used as indicators of agronomic performance of the cropping systems evaluated in this study. The results showed that all the agronomic parameters except the soybean chlorophyll contents were significantly affected by IC corn with forage soybeans (Tables 3–5).

**Table 1:** The description of experimental treatments during both growing seasons.

Treatment	Cropping System	Genotypes
C1	Corn-monocropping	Yukon-R
C2	Corn-monocropping	DKC26-28RIB
S1	Soybean-monocropping	Big Fellow-RR (upright)
S2	Soybean-monocropping	Game Keeper (upright)
S3	Soybean-monocropping	Kester’s Bob White Trailing Soybeans (vine type)
S1C1	Intercropping	Big Fellow RR + Yukon-R
S2C1	Intercropping	Game Keeper RR + Yukon-R
S3C1	Intercropping	Kester’s Bob White Trailing Soybeans + Yukon-R
S1C2	Intercropping	Big Fellow RR + DKC26-28RIB
S2C2	Intercropping	Game Keeper RR + DKC26-28RIB
S3C2	Intercropping	Kester’s Bob White Trailing Soybeans + DKC26-28RIB

**Table 2:** Phospholipid fatty acids (PLFA) biomarkers used to characterize the active microbial community structure.

Fatty Acids	Organisms	Reference
2OH-C10:0	G−	^30^
C14:0	G+	^51^
C14:1n-5	G−	^52^
i-C15:0	G+	^52, 53^
a-C15:0	G+	^52, 53^
C15:0	G+	^54, 55^
i-C16:0	G+	^52, 53^
2OH-C12:0	G−	^30^
C16:0	G+ & G−	^56, 57^
C16:1n-7	G+ & G−	^53, 58^
i-C17:0	G+	^52, 53^
3OH-C12:0	G−	^59^
C17:0	G+	^54, 55^
C17:1n-7	G−	^60^
cyclo-C17:0	G−	^52, 53^
C18:0	G+ & G−	^57, 58^
C18:1n-9trans	G−	^61^
C18:1n-9cis	G+ & G− & F	^52, 58^
3OH-C14:0	G−	^54^
C18:2n-6cis	F	^52, 62^
C18:3n-3	F	^41, 57, 63, 64^
Cyclo-C19:0	G−	^53^
2OH-C16:0	G−	^51^
C20:0	P	^65^
20:1n-9cis	F	^41, 63^
20:3n-6	P	^66^
C20:4n-6	P	^67^

G+: gram positive bacteria; G−: gram negative bacteria; F: fungi; P: protozoa.

**Table 3:** Chlorophyll contents of silage corn and soybean plants cultivated as either monocrop or intercropped during the growing seasons.

Treatments	Growing Season (2016–17)	
	Corn	Soybean
C1	42.53 ± 1.20^bc^	—
C2	41.17 ± 1.57^c^	—
S1	—	31.10 ± 1.77
S2	—	31.66 ± 1.67
S3	—	29.77 ± 1.38
S1C1	45.03 ± 1.41^ab^	27.55 ± 1.54
S2C1	46.19 ± 1.12^a^	27.34 ± 1.85
S3C1	44.69 ± 1.21^ab^	28.73 ± 1.36
S1C2	46.07 ± 0.71^a^	27.97 ± 1.83
S2C2	46.67 ± 0.40^a^	28.45 ± 1.35
S3C2	43.88 ± 1.00^abc^	27.63 ± 1.34
Mono-(US)	31.38 ± 1.16^A^	
Inter -(US)	27.83 ± 0.78^B^	
Mono-(VS)	29.77 ± 1.38	
Inter-(VS)	28.18 ± 0.92	
Mono-C	41.85 ± 0.97^B^	
Inter-C	45.42 ± 0.41^A^	
Mono-S	30.85 ± 0.90^A^	
Inter-S	28.01 ± 0.60^B^	

Values are means ± standard errors. Mean values in each column followed by the same superscripts are not significantly different at alpha 0.05. C: corn; S: soybean; C + S: corn + soybean; C1: Yukon-R; C2: DKC26-28RIB; S1: Big Fellow RR; S2: Game Keeper RR; S3: Kester’s Bob White Trailing Soybean; S1C1: Big Fellow RR + Yukon-R; S2C1: Game Keeper RR + Yukon-R; S3C1: Kester’s Bob White Trailing Soybean + Yukon-R; S1C2: Big Fellow RR + DKC26-28RIB; S2C2: Game Keeper RR + DKC26-28RIB; S3C2: Kester’s Bob White Trailing Soybean + DKC26-28RIB; Mono-C: monocropping corn; Mono-S: monocropping soybean; Inter-S: intercropped soybean; Inter-C: corn intercropped with soybean; Mono-(US): monocropping upright soybean; Mono-(VS): monocropping vine soybean; Inter-(US): intercropped upright soybean; Inter-(VS): intercropped vine soybean.

**Table 4:** Plant height (cm) of silage corn and forage soybean either monocropped or intercropped during the growing seasons.

Treatments	Growing season (2016–17)	
	Corn	Soybean
C1	199.67 ± 1.58^ab^	—
C2	181.80 ± 2.97^d^	—
S1	—	54.80 ± 1.68^c^
S2	—	52.90 ± 1.60^cd^
S3	—	124.97 ± 1.79^a^
S1C1	206.57 ± 2.16^a^	50.70 ± 1.81^cde^
S2C1	204.83 ± 2.76^a^	48.67 ± 1.71^de^
S3C1	206.00 ± 1.59^a^	111.67 ± 2.79^b^
S1C2	194.30 ± 4.35^bc^	51.83 ± 1.69^cd^
S2C2	188.67 ± 1.61^cd^	45.93 ± 1.37^e^
S3C2	189.80 ± 3.41^c^	111.53 ± 1.97^b^
Mono-(US)	53.85 ± 1.14^A^	
Inter-(US)	49.28 ± 0.90^B^	
Mono-(VS)	124.97 ± 1.79^A^	
Inter-(VS)	111.60 ± 1.63^B^	
Mono-S	77.56 ± 8.18	
Inter-S	70.79 ± 5.05	
Mono-C	190.73 ± 3.13^B^	
Inter-C	198.36 ± 1.68^A^	

Values are means ± standard errors. Mean values in each column followed by the same superscripts are not significantly different at alpha 0.05. C: corn; S: soybean; C + S: corn + soybean; C1: Yukon-R; C2 = DKC26-28RIB; S1: Big Fellow RR; S2: Game Keeper RR; S3: Kester’s Bob White Trailing Soybean; S1C1: Big Fellow RR + Yukon-R; S2C1: Game Keeper RR + Yukon-R; S3C1: Kester’s Bob White Trailing Soybean + Yukon-R; S1C2: Big Fellow RR + DKC26-28RIB; S2C2: Game Keeper RR + DKC26-28RIB; S3C2 Kester’s Bob White Trailing Soybean + DKC26-28RIB; Mono-C: monocropping corn; Mono-S: monocropping soybean; Inter-S: intercropped soybean; Inter-C: corn intercropped with soybean; Mono-(US): monocropping upright soybean; Mono-(VS): monocropping vine soybean; Inter-(US): intercropped upright soybean; Inter-(VS): corn intercropped with vine soybean.

**Table 5:** Forage production (Mg ha^−1^) of corn and soybean either monocropped or intercropped during the growing seasons.

Treatments	Growing Season (2016–17)		
	C	S	C + S
C1	12.04 ± 1.03	—	12.04 ± 1.03^bc^
C2	11.09 ± 1.12	—	11.09 ± 1.12^c^
S1	—	3.57 ± 0.12^a^	3.57 ± 0.12^d^
S2	—	3.36 ± 0.18^a^	3.36 ± 0.18^d^
S3	—	1.05 ± 0.16^b^	1.05 ± 0.16^e^
S1C1	14.23 ± 0.75	1.12 ± 0.07^b^	15.35 ± 0.75^a^
S2C1	14.30 ± 0.88	1.13 ± 0.10^b^	15.43 ± 0.95^a^
S3C1	13.86 ± 0.51	0.43 ± 0.08^c^	14.29 ± 0.58^a^
S1C2	13.58 ± 0.70	0.96 ± 0.11^b^	14.54 ± 0.80^a^
S2C2	14.06 ± 1.24	1.07 ± 0.11^b^	15.12 ± 1.24^a^
S3C2	13.47 ± 0.61	0.35 ± 0.07^c^	13.82 ± 0.68^ab^
Mono-(US)		3.46 ± 0.11^A^	
Inter -(US)		1.07 ± 0.05^B^	
Mono-(VS)		1.05 ± 0.16^A^	
Inter-(VS)		0.39 ± 0.05^B^	
Mono-C		11.57 ± 0.74^B^	
Inter-C		13.92 ± 0.31^A^	
Mono-S		2.66 ± 0.29^c^	
Mono-C		11.57 ± 0.74^B^	
Inter-(C + S)		14.76 ± 0.34^A^	

Values are means ± standard errors. Mean values in each column followed by the same superscripts are not significantly different at alpha 0.05. C: corn; S: soybean; C + S: corn + soybean; C1: Yukon-R; C2: DKC26-28RIB; S1: Big Fellow RR; S2: Game Keeper RR; S3: Kester’s Bob White Trailing Soybean; S1C1: Big Fellow RR + Yukon-R; S2C1: Game Keeper RR + Yukon-R; S3C1: Kester’s Bob White Trailing Soybean + Yukon-R; S1C2 = Big Fellow RR + DKC26-28RIB; S2C2: Game Keeper RR + DKC26-28RIB; S3C2 Kester’s Bob White Trailing Soybean + DKC26-28RIB; Mono-C: monocropping corn; Mono-S: monocropping soybean; Inter-S: intercropped soybean; Inter-C: corn intercropped with soybean; Mono-(US): monocropping upright soybean; Mono-(VS): monocropping vine soybean; Inter-(US): intercropped upright soybean; Inter-(VS): intercropped vine soybean.

In general, the chlorophyll contents of corn intercropped with soybean significantly increased compared to corn MC, while a reduction in the soybean plant chlorophyll contents was observed after IC with corn (Table 3). Upright soybean (US) varieties had significant decreased chlorophyll contents when intercropped with corn compared to the vine type soybean (VS) chlorophyll contents. The highest corn chlorophyll contents were measured in S2C2 (46.67), while the lowest was measured in C2 (41.17). For soybean, the highest chlorophyll contents were observed in S2 (31.66), while the lowest was observed in S2C1 (27.34).

The overall average corn plant height was significantly different between the monocropped and intercropped treatments (Table 4). However, we observed a significant reduction in the soybean plant height when both upright and vine type soybeans were intercropped with corn (Table 4). In general, the plant height in upright soybeans MC was 54 cm compared to 49 cm in the IC. Conversely, the plant height of S3 (vine soybean) cultivated as MC was 125 cm compared to 112 cm in the IC treatments (Table 4). The trend was opposite for corn plants when compared to soybeans. IC significantly (p < 0.05) increased the corn plant height as compared to MC treatments (Table 4). The highest and lowest corn plant heights were recorded in S1C1 (206.57 cm) and C2 (181.80 cm), respectively during the growing season.

In general, IC treatments significantly increased the total FP as compared to corn and forage soybean MC (Table 5). Overall, IC treatments produced 28% higher FP compared to corn MC treatments. It is important to note that the S3 treatment is a vine soybean that were intercropped with silage corn. Overall, IC increased the corn FP but decrease the FP of soybean during the growing seasons (Table 5).

The highest FP was observed in the S2C1 (15.43 Mg ha^−1^) IC treatment, while the lowest yields were observed in C2 (11.09 Mg ha^−1^) and S3 (1.05 Mg ha^−1^) MC treatments. The LER values were greater than 1 for all IC treatments compared to MC treatments during both years (Supplementary Table S2), that means, more land is required in MC to produce the same crop yield as was obtained in the IC treatments. The observed LER values for the IC treatments ranged from 1.49–1.58 (Supplementary Table S2).

#### Rhizosphere soil acid phosphatase activity

**Figure 2:**
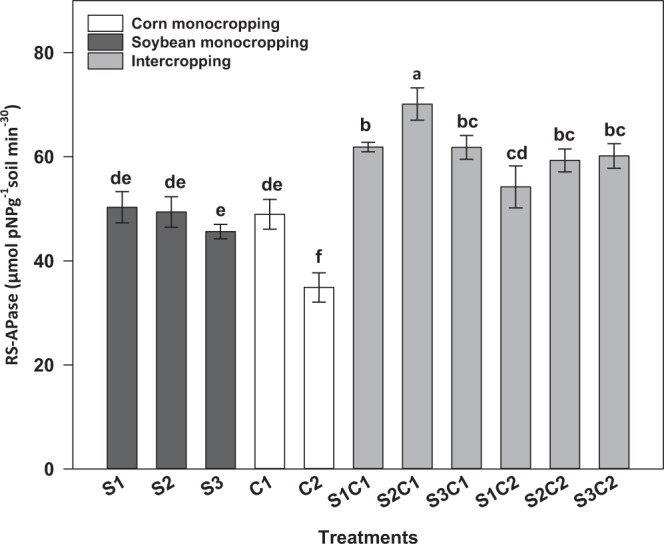
Rhizosphere soil acid phosphates (RS-APase) activity of corn and soybean sown as monocrops and as intercropping (n = 66). The error bar represents means ± SE of combined data collected in 2016 and 2017 field seasons. Different letters indicate significant differences at alpha 0.05 between treatments. C1: Yukon-R; C2: DKC26-28RIB; S1: Big Fellow RR; S2: Game Keeper RR; S3: Kester’s Bob White Trailing Soybean; S1C1: Big Fellow RR + Yukon-R; S2C1: Game Keeper RR + Yukon-R; S3C1: Kester’s Bob White Trailing Soybean + Yukon-R; S1C2: Big Fellow RR + DKC26-28RIB; S2C2: Game Keeper RR + DKC26-28RIB; S3C2: Kester’s Bob White Trailing Soybean + DKC26-28RIB.

**Figure 3:**
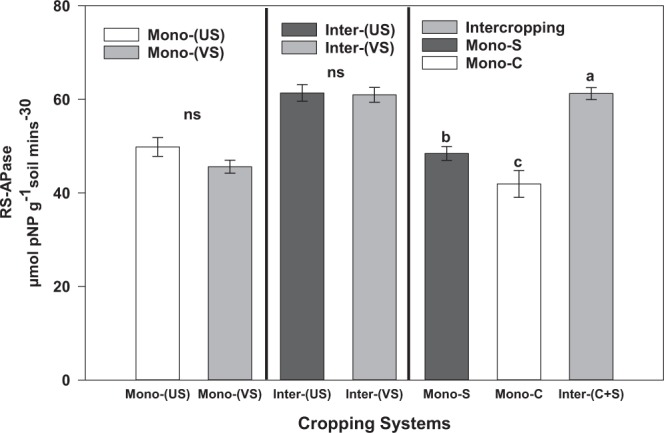
Rhizosphere soil acid phosphatase (µmole pNP g^−1^ soil 30 min^−1^) activity of corn and soybean cropping systems. The error bar represents means ± SE of combined data collected in 2016 and 2017 field seasons. Different letters indicate significant differences between the cropping systems at alpha 0.05. Mono-(US): monocropping of upright soybean; Mono-(VS): monocropping vine soybean; Inter-(US): intercropped upright soybean; Inter-(VS): intercropped vine soybean; Mono-C: monocropping corn; Mono-S: monocropping soybean.

The (RS-APase) activity was significantly higher (p < 0.05) in the IC treatments compared to MC (Fig. 2). However, no significant difference was observed in (RS-APase) activity between upright (S1 and S2) and vine (S3) soybean varieties in either IC or MC treatments (Fig. 3). The highest RS-APase was observed in S2C1 (70.13 µmol pNP g^−1^ soil 30 min^−1^). Conversely, the lowest RS-APase was recorded in C2 (34.90 µmol pNP g^−1^ soil 30 min^−1^).

### Changes in rhizosphere available P and pH

The changes in RS-P_available_ and RS-pH due to different IC treatments are given in Table 6. There was no difference in RS-P_available_ or pH between the VS and US cultivated as MC. However, when both the US and VS were intercropped with corn, there was a significant increase in the content of RS-P_available_ compared to the MC treatments.

**Table 6:** Rhizosphere soil available phosphorus and pH of silage corn and forage soybean cultivated as monocropp and intercrop during the growing seasons.

Treatments	Growing Season (2016–17)	
	RS-P_available_	RS-pH
C1	46.70 ± 1.94^e^	5.58 ± 0.08
C2	41.27 ± 1.26^e^	5.63 ± 0.18
S1	76.16 ± 5.94^b^	5.79 ± 0.14
S2	45.52 ± 3.80^e^	5.89 ± 0.27
S3	65.54 ± 1.61^cd^	5.78 ± 0.23
S1C1	81.78 ± 1.82^ab^	5.27 ± 0.07
S2C1	81.87 ± 3.23^ab^	5.44 ± 0.26
S3C1	60.13 ± 2.31^d^	5.67 ± 0.21
S1C2	91.44 ± 5.33	5.61 ± 0.20
S2C2	74.65 ± 5.36^bc^	5.49 ± 0.25
S3C2	72.88 ± 2.60^bc^	5.48 ± 0.17
Mono-(US)	60.84 ± 5.71	5.84 ± 0.15
Mono-(VS)	65.54 ± 1.61	5.78 ± 0.23
Inter-(US)	82.44 ± 2.32^A^	5.45 ± 0.10
Inter-(VS)	66.50 ± 2.54^B^	5.57 ± 0.13
Mono-S	62.41 ± 3.82^B^	5.82 ± 0.12^A^
Mono-C	43.99 ± 1.37^C^	5.61 ± 0.09^AB^
Inter-(C + S)	77.12 ± 2.16^A^	5.49 ± 0.08^B^

Values are means ± standard errors. Mean values in each column followed by the same superscripts are not significantly different at alpha 0.05. C: corn; S: soybean; C + S: corn + soybean; C1: Yukon-R; C2: DKC26-28RIB; S1: Big Fellow RR; S2: Game Keeper RR; S3: Kester’s Bob White Trailing Soybean; S1C1: Big Fellow RR + Yukon-R; S2C1: Game Keeper RR + Yukon-R; S3C1: Kester’s Bob White Trailing Soybean + Yukon-R; S1C2: Big Fellow RR + DKC26-28RIB; S2C2: Game Keeper RR + DKC26-28RIB; S3C2: Kester’s Bob White Trailing Soybean + DKC26-28RIB; Mono-C: monocropping corn; Mono-S: monocropping soybean; Inter(C + S): corn-soybean intercropping; Mono-(US): monocropping upright soybean; Mono-(VS): monocropping vine soybean; Inter-(US): intercropped upright soybean; Inter-(VS): intercropped vine soybean. Soil available phosphorus: RS-P_available_.

The increase in RS-P_available_ was significantly greater in the US varieties intercropped with corn, than when IC was done with the VS. Furthermore, the increase in RS-P_available_ was significantly (p < 0.05) different for the IC treatments compared to the corn MC treatments. The highest RS-P_available_ was measured in S1C2 (91.44 mg kg^−1^), and the lowest in C2 (41.27 mg kg^−1^). Similarly, the highest P availability was observed in S1C2 (102.71 mg kg^−1^), but the lowest was in S2 (37.66 mg kg^−1^).

Overall corn and soybean IC decreased the RS-pH as compared to when cultivated as MC, but the decrease was not significant. Similarly, there was no significant difference observed between RS-pH of US or VS cultivated as monocrops. The highest pH was measured in S2 (5.89), and the lowest in S1C1 (5.27) of all treatments evaluated in this study (Table 6).

### Soil microbial community composition

The effect of corn-soybean IC on rhizosphere active soil microbial community structure (gram-positive (G+) or gram-negative (G−) bacteria, fungi, and protozoa) was assessed using microbial membrane PLFAs (Tables 2 and 7). The total bacterial population was significantly higher in all treatments than the fungi and protozoa population. Both G+ and G− bacteria contributed (48% and 52%, respectively) to the overall observed total bacterial population. Overall, the active microbial population (G+, G− and protozoa) was significantly (p < 0.05) higher in IC compared to the corn and soybean MC; except for the fungal population which was not significantly different than that of the corn MC treatments (Table 7). The overall trend showed that the microbial community biomass was higher when corn was intercropped with upright soybean varieties than when intercropped with vine soybean. The highest G+ and G− bacterial populations were found in S1C1 (27.83 nmol g^−1^), while the lowest population was found in S2 (20.66 nmol g^−1^) treatment. The fungi and protozoa populations also followed similar trends. The highest populations were observed in S1C1 (7.45 and 1.86 nmol g^−1^), while the lowest populations of both fungi and protozoans were observed in S3 (4.57, 1.52 nmol g^−1^) treatment respectively. The maximum G+: G− and fungi: bacteria ratios were recorded in C2, but overall these ratios were not significantly different between the MC and IC treatments (Table 7). The total PLFAs content indicate the overall active microbial population was higher in IC treatments compared to corn and soybean MC treatments.

**Table 7:** The sum of selected PLFAs (nmol g^−1^) used to assess the active microbial community structure in the rhizosphere of corn and soybean cultivated as either monocrop or intercrop during the growing seasons.

Treatments	G+	G−	B	F	P	T (PLFAs)	G+: G−	F: B
C1	21.02 ± 0.30^d^	22.50 ± 0.46^c^	43.52 ± 0.75^d^	4.76 ± 0.14^cd^	1.63 ± 0.03^cde^	49.90 ± 0.89^d^	0.94 ± 0.01	0.11 ± 0.00^c^
C2	21.41 ± 1.03^d^	22.81 ± 1.43^c^	44.23 ± 2.44^d^	5.79 ± 0.59^b^	1.62 ± 0.06^cde^	51.63 ± 3.07^cd^	0.94 ± 0.02	0.13 ± 0.01^a^
S1	21.36 ± 0.45^d^	23.23 ± 0.68^c^	44.69 ± 1.12^d^	5.16 ± 0.38^bcd^	1.63 ± 0.06^cde^	51.49 ± 1.53^d^	0.92 ± 0.01	0.11 ± 0.01^bc^
S2	20.66 ± 0.14^d^	22.32 ± 0.22^c^	42.98 ± 0.27^d^	4.96 ± 0.11^bcd^	1.55 ± 0.02^e^	49.49 ± 0.31^d^	0.93 ± 0.01	0.12 ± 0.00^abc^
S3	20.94 ± 0.56^d^	22.64 ± 0.47^c^	43.58 ± 0.98^d^	4.57 ± 0.15^d^	1.52 ± 0.01^e^	49.68 ± 0.95^d^	0.93 ± 0.01	0.11 ± 0.00^c^
S1C1	27.83 ± 1.47^a^	29.58 ± 1.42^a^	57.41 ± 2.89^a^	7.45 ± 0.74^a^	1.86 ± 0.06^a^	66.72 ± 3.66^a^	0.94 ± 0.01	0.13 ± 0.01^ab^
S2C1	23.81 ± 0.64^bc^	26.22 ± 1.03^b^	50.02 ± 1.66^bcd^	5.61 ± 0.36^bcd^	1.74 ± 0.07^abc^	57.37 ± 1.99^bc^	0.91 ± 0.01	0.11 ± 0.00^c^
S3C1	21.96 ± 0.75^cd^	23.50 ± 0.79^c^	45.46 ± 1.52^cd^	4.67 ± 0.17^cd^	1.60 ± 0.02^de^	51.73 ± 1.58^cd^	0.93 ± 0.01	0.10 ± 0.00^e^
S1C2	24.41 ± 1.25^b^	26.21 ± 1.48^b^	50.63 ± 2.72^b^	5.75 ± 0.41^bc^	1.79 ± 0.07^ab^	58.17 ± 3.18^b^	0.93 ± 0.01	0.11 ± 0.00^c^
S2C2	21.37 ± 0.48^d^	23.39 ± 0.48^c^	44.76 ± 0.91^d^	4.68 ± 0.13^cd^	1.66 ± 0.01^cde^	51.10 ± 0.90^d^	0.91 ± 0.01	0.10 ± 0.00^c^
S3C2	22.13 ± 0.44^cd^	24.10 ± 0.60^bc^	46.22 ± 0.95^bcd^	5.28 ± 0.32^bcd^	1.71 ± 0.03^bcd^	53.20 ± 1.13^bcd^	0.92 ± 0.02	0.11 ± 0.01^c^
Average	22.45 ± 0.33	24.24 ± 0.37	46.68 ± 0.70	5.33 ± 0.14	1.66 ± 0.02	53.68 ± 0.84	0.93 ± 0.00	0.11 ± 0.00
Mono-(US)	21.01 ± 0.25	22.83 ± 0.37	43.84 ± 0.61	5.06 ± 0.19	1.59 ± 0.03	50.49 ± 0.80	0.92 ± 0.01	0.12 ± 0.00
Mono-(VS)	20.94 ± 0.56	22.64 ± 0.47	43.58 ± 0.98	4.57 ± 0.15	1.52 ± 0.01	49.68 ± 0.95	0.93 ± 0.01	0.11 ± 0.00
Inter-(US)	24.35 ± 0.68^A^	26.35 ± 0.71^A^	50.71 ± 1.39^A^	5.87 ± 0.30	1.76 ± 0.03^A^	58.34 ± 1.70^A^	0.92 ± 0.01	0.11 ± 0.00
Inter-(VS)	22.04 ± 0.41^B^	23.80 ± 0.48^A^	45.84 ± 0.86^A^	4.97 ± 0.20	1.65 ± 0.02^B^	52.47 ± 0.95^B^	0.93 ± 0.01	0.11 ± 0.00
Mono-S	20.99 ± 0.24^B^	22.76 ± 0.29^B^	43.75 ± 0.50^B^	4.90 ± 0.14^B^	1.57 ± 0.02^B^	50.22 ± 0.61^B^	0.92 ± 0.01	0.11 ± 0.00
Mono-C	21.22 ± 0.51^B^	22.66 ± 0.72^B^	43.87 ± 1.22^B^	5.27 ± 0.33^AB^	1.62 ± 0.03^B^	50.77 ± 1.55^B^	0.94 ± 0.01	0.12 ± 0.00
Inter (C + S)	23.58 ± 0.51^A^	25.50 ± 0.54^A^	49.08 ± 1.04^A^	5.57 ± 0.22^A^	1.73 ± 0.02^A^	56.38 ± 1.26^A^	0.93 ± 0.01	0.11 ± 0.00

Values are means ± standard errors. Mean values in each column followed by the same superscripts are not significantly different at alpha 0.05. C: corn; S: soybean; C + S: corn + soybean; C1: Yukon-R; C2: DKC26-28RIB; S1: Big Fellow RR; S2: Game Keeper RR; S3: Kester’s Bob White Trailing Soybean; S1C1: Big Fellow RR + Yukon-R; S2C1: Game Keeper RR + Yukon-R; S3C1: Kester’s Bob White Trailing Soybean + Yukon-R; S1C2: Big Fellow RR + DKC26-28RIB; S2C2: Game Keeper RR + DKC26-28RIB; S3C2: Kester’s Bob White Trailing Soybean + DKC26-28RIB; Mono-C: monocropping corn; Mono-S: monocropping soybean; Inter-(C + S): corn-soybean intercropping; Mono-(US): monocropping upright soybean; Mono-(VS): monocropping vine soybean; Inter-(US): intercropped upright soybean; Inter-(VS): intercropped vine soybean. G+: gram positive; G−: gram negative; B: bacteria; T (PLFAs): total phospholipid fatty acids; F: fungi; P: protozoa.

### Relationship between soil health status and agronomic performance following corn-soybean cultivation under cool climatic conditions in boreal ecosystem

**Figure 4:**
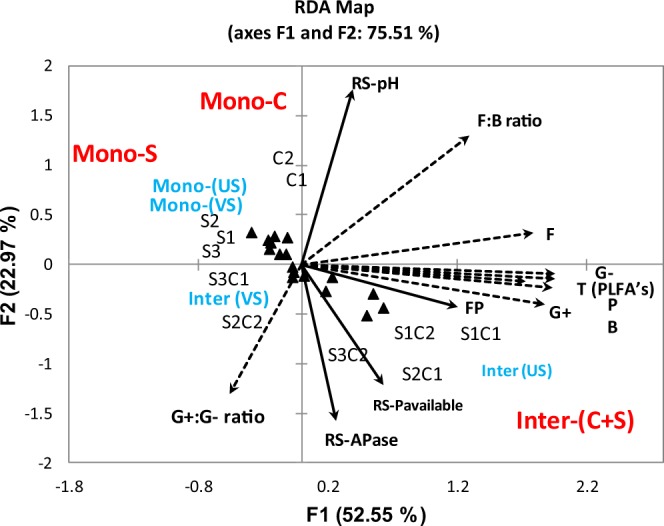
Redundancy analysis (RDA) of the active soil microbial community (PLFA), soil chemical properties and forage production in corn soybean intercropping treatments. RS-APase, RS-P_available_, and FP represent rhizosphere soil acid phosphatase activity, rhizosphere soil available phosphorus and forage production respectively. G+, G−, B, T PLFAs, F, P represent gram positive, gram negative, bacteria, total phospholipid fatty acids, fungi, and protozoa respectively. Mono-C: monocropping corn; Mono-S: monocropping soybean; Inter-(C + S): corn-soybean intercropping; Mono-(US): monocropping upright soybean; Mono-(VS): monocropping vine soybean; Inter-(US): intercropped upright soybean; Inter-(VS): intercropped vine soybean.

**Figure 5:**
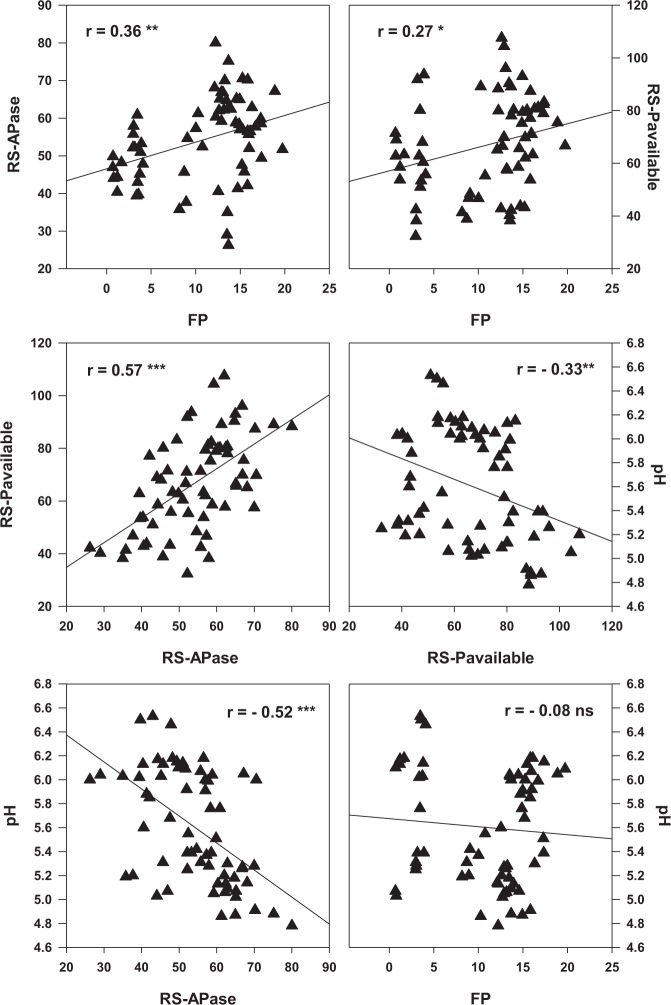
Pearson correlations showing the association between rhizosphere acid phosphatase activity (RS-APase: µmol g^−1^soil 30 min^−1^), rhizosphere available phosphorus (RS-P_available_: mg kg^−1^), soil pH and forage production (FP: Mg ha^−1^) for corn soybean monocropping and intercropping treatments. ns = correlation is not significant; *Correlation is significant (p ≤ 0.05); (**p ≤ 0.01); (***p ≤ 0.001) and *n* is 66 for all parameters.

**Figure 6:**
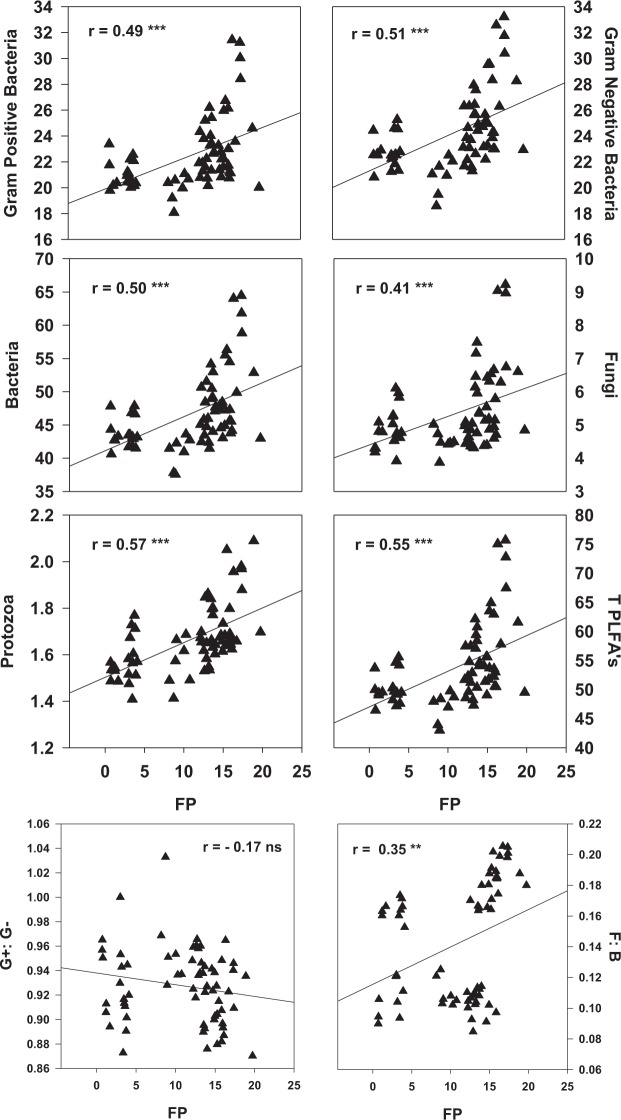
Pearson correlations showing the association between forage production (FP: Mg ha^−1^) and rhizosphere soil microbial community for corn soybean monocropping and intercropping treatments. T PLFAs = total phospholipid fatty acids; G+: G− = gram positive to gram negative bacteria ratio; F: B = fungi to bacteria ratio; FP = forage production; ns = correlation is not significant; *Correlation is significant (p ≤ 0.05); (**p ≤ 0.01); (***p ≤ 0.001) and *n* is 66 for all parameters. PLFAs were measured as nmol g^−1^ soil.

**Figure 7:**
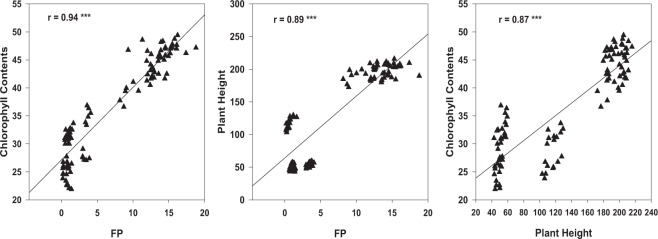
Correlation between plant height (cm), chlorophyll contents and forage production (Mg ha^−1^) for the different corn soybean monocropping and intercropping treatments. ns = correlation is non-significant; *Correlation is significant (p ≤ 0.05); (**p ≤ 0.01); (***p ≤ 0.001) n = 66 for all parameter.

The active microbial community structure, RS-pH, RS-APase activity, and RS-P_available_ were used as indicators to assess the soil health status; while the chlorophyll contents, final plant height and FP were used as indicators of agronomic performance (Figs 4–7; Table 8).

**Table 8:** Pearson correlation coefficients between phospholipid fatty acids (PLFAs) and rhizosphere soil acid phosphates activity (RS-APase) and rhizosphere soil available phosphorus (RS-P_availlable_).

	G+	G−	B	F	P	T PLFAs	G+: G−	F: B
**2016–17**								
RS-APase	0.21^ns^	0.16^ns^	0.18^ns^	0.18^ns^	0.14^ns^	0.14^ns^	0.17^ns^	−0.38**
RS-P_availlable_	0.32**	0.31**	0.32**	0.09^ns^	0.31**	0.29*	−0.01^ns^	−0.19^ns^

ns: correlation is non-significant; *Correlation is significant (p ≤ 0.05); (**p ≤ 0.01); (***p ≤ 0.001). *n* = 66.

Several significant associations were observed between the active microbial community structure, soil biochemical properties and agronomic performance (Figs 5–7; Table 8). These associations were very influential in clustering the MC treatments in different quadrants of the biplot from the IC treatments following redundancy analysis (Fig. 4). For example, RS-APase, RS-P_available_ and FP were the most influential factors clustering the IC together (Fig. 4). We also observed that vine type forage soybean intercropped with silage corn [Inter-(VS)] clustered in separate quadrants of the biplots compared to US intercropped with silage corn [Inter (US)]. The output from the RDA analysis revealed that axis 1 and axis 2 explained 52.55% and 22.97% of the total variance (Fig. 4). Significant positive correlations were observed between the soil microbial PLFAs biomarkers (G+, G−, P, F, T PLFAs), RS-P_available_ and FP. Conversely, all these parameters were negatively correlated with RS-pH (Figs 5 and 6; Table 8). Furthermore, the G+: G− and F:B ratios were negatively correlated with RS‒APase, RS-P_available_ and FP; except for the F:B ratio which showed a positive correlation with FP (Fig. 4). Further analysis by Pearson correlation confirmed the significant associations between the active soil microbes, RS-P_available_, and FP (Figs 5 and 6, Table 8). For example, FP was observed to be significantly associated with not only RS-APase activity and RS-P_available_ but also the following estimated soil microbial PLFAs biomarkers (B, P, F:B, G+: G-, total PLFAs). However, FP was inversely associated with the RS-pH. A consistent pattern also emerged that indicates the RS-APase activity was significantly associated with the RS-P_available_ (r = 0.57) of podzols present in cool climate boreal ecosystems. The RS-P_available_ was also observed to be significantly associated with most of the active soil microbial population present in the root rhizosphere, except for fungi (Table 8). The agronomic parameters (plant height, chlorophyll content and forage production) were also significantly, and positively correlated (r = 0.87–0.94) with each other (Fig. 7). Specifically, the chlorophyll content was highly associated with increase forage production. This trend was similar between plant height and total forage production in the inter and mono cropping systems evaluated in this study.

## Discussion

The growing seasons in cool climate boreal environments are short and characterized by low growth temperatures averaging between 14–17 °C^3^, consistent with the low growth temperatures observed in Newfoundland during the study (Fig. 1). In the current study, we attempted to evaluate the effects of intercropping silage corn with vine type or upright forage soybeans on the crop agronomic performance and soil health status following field cultivation under cool climatic conditions in boreal ecosystems. Intercropping (IC) can enhance crop growth and productivity due to superior utilization of above and below ground resources^4,44^. Plant chlorophyll content is an important indicator of agronomic performance (enhanced crop growth and productivity) and play a key role in plant photosynthesis^45^. Chlorophyll content and plant height of corn plants increased (by 8.5% and 4% respectively) in the IC treatments compared to MC (Tables 3 and 4). Conversely, the soybean chlorophyll content and plant height decreased (−9.4% and −9.7% respectively) in the IC treatments compared to the MC treatments (Tables 3 and 4). These findings are in agreement with previous studies reported in the literature^46–48^, and suggest that both the chlorophyll content and plant height could be the possible drivers for the changes observed in forage production. This is consistent with the high correlations observed between chlorophyll content, plant height and total forage production (Fig. 7).

IC can result in either increased forage production (LER > 1), decreased forage production (LER < 1), or have no effects on forage production (LER = 1)^12,49^. In our experiments, IC increased the FP compared to either corn or soybean MC following cultivation on podzols under cool climatic conditions in boreal ecosystems. The increase observed in FP suggested that IC has an advantage over MC in terms of plant growth, which is also supported by the LER values which were greater than 1 in all IC treatments; further confirming that corn and soybean IC in cool climate boreal environment production systems was superior compared to MC in regards to increasing FP (Supplementary Table S2). Our LER findings are consistent with the results obtained by several other researchers who reported LER values greater than 1 in IC following crop production under warmer climatic conditions^14,23,50^. The LER values in our study suggest that 49–58% more land would be required for the MC production system to produce a crop yield equal to that of the IC production system in boreal ecosystem. These findings indicate that the IC system appears to use land resources (nutrients) more efficiently than the MC system^17^, and this may account for the superior agronomic performance (enhanced forage production) observed when the crops were cultivated under cool climatic conditions in boreal ecosystem.

The study results demonstrated that IC increased the RS-APase activity between 46% and 26% compared to corn and soybean MC treatments, respectively (Fig. 2). These findings suggest IC may utilize organic P more efficiently than MC corn when cultivated on podzols under cool climatic conditions in boreal ecosystem. Organic P comprises 30–80% of total P in most agricultural soils, and can be converted into RS‒P_available_ forms after hydrolyzation by phosphate enzymes^51,52^, consistent with the high association observed between RS‒P_available_ and the RS-APase activity (Figs 4 and 5) in this study. The higher RS-APase activity in IC could also be attributed to compatibility or suitability of the silage corn and soybean combinations as companion plants in an IC production system under cool climatic conditions. In arid soils, the RS‒P_available_ was significantly correlated with RS‒APase activity^53^ because of an association between mobilization of organic P and RS‒APase activity^54,55^. Our results are similar with this and other studies, which reported that cereal-legume IC increased the RS-APase activity compared to when either silage corn or forage soybean were cultivated as monocrops^7,47^. The legume species have been considered as the major contributor to the increased RS‒APase activity observed in IC due to the fact that large amounts of acid phosphatase are known to be released from their roots into the root rhizosphere^26,56^. This may account in part for the increase forage production observed in IC considering the significant correlations observed between RS‒APase activity, the RS‒P_available_ and forage production (Fig. 5).

Rhizosphere acidification have been previously reported in several IC systems following cultivation in warm climate production systems^7,12,28,57^. Similar to these findings, we observed that IC decreased the RS-pH compared to when corn and soybean were cultivated as monocrops under cool climatic conditions in boreal ecosystem (Table 6). The rhizosphere acidification was shown in previous studies to be due to the release of large quantities of protons or organic acids in the root rhizosphere from the crop roots during IC^28,58^. Similar to the RS‒pH, IC had positive effects on RS-P_available_ when silage corn and forage soybeans are cultivated as the companion crops in cool climate boreal ecosystem. In fact, IC increased the RS-P_available_ between 74% and 26% in the plant root rhizosphere compared to when corn and soybeans were cultivated as monocrops (Table 6). Consistent with our findings, increased P availability in the rhizosphere have also been reported in intercropping garlic-cucumber^59^; and maize-chickpea^26^. Increase in RS-APase activity and acidification of the rhizosphere via release of protons and organic acids have been suggested to be responsible for the enhanced RS‒P_available_ observed in IC production systems^28^; and maybe related to the significant inverse relationships observed in our study between RS‒P_available_ or RS-APase activity and RS‒pH (Fig. 5).

The active microbial community was investigated by performing PLFAs analysis present in the root rhizosphere following corn and soybean cultivation as either mono or intercrops in podzolic soils under cool climatic conditions in boreal ecosystem. PLFAs are present in the membranes of living cells, but not in dead cells because of rapid degradation during cell death. As such, they can give an accurate estimate of the living (active) microbial community present in the root rhizosphere, and how these community composition change in response to factors such as crop management systems, environmental conditions, and production inputs^60^. Different microbial groups present in the soil are comprised of phospholipid fatty acids in their membranes that are diagnostics of their presence and rate of change in the soil habitat^61^. Consequently, the diversity of the active microbial community is referred to as an imperative indicator of soil quality or the health status of the soil^62^. As such, a combination of PLFA markers were used consistent with convention in the literature to delineate changes in the microbial community composition or structure in response to the cropping systems evaluated in this study. Our results showed that IC in general, increased the total microbial PLFAs biomarkers (G+, G−, F and P) in the root rhizosphere as compared to soybean and corn MC. This increase was 12.3% and 12.3% (Table 7). Our results corroborate the findings of Li *et al*.^41^ and Zhou *et al*.^4^ who demonstrated that IC can enhance both bacterial and fungal populations in the plant root rhizosphere. However, these studies were not conducted on crops cultivated in cool climate production systems. To our knowledge, this is the first work demonstrating the potential of intercropping to modulate the active microbial population in podzolic soils present in cool climate boreal ecosystems. We observed that the bacterial population was the highest of the total PLFAs biomarkers present in corn and soybeans cultivated as MC or IC under cool climatic conditions in boreal ecosystem (Table 7). However, the G− population was 4% higher than the G+ bacterial population (Table 7). Higher proportion of G+ bacterial population compared to G− suggested a deficiency of organic carbon in the soil^63,64^. In contrast, the dominance of G− bacteria over G+ bacteria in the soil, and a high fungal population is characteristic of the presence of higher amount of complex organic matter in the soil^63,65^. The findings from this work demonstrate that corn soybean IC promoted the growth and diversity of the active microbial community, and as such can enhance the soil health status under cool climatic conditions in boreal ecosystems.

The significant positive correlation observed between the agronomic and soil chemical properties demonstrated that these parameters are associated with the superior FP (Figs 6 and 7; Table 8) observed when corn was IC with soybean and cultivated under cool climatic conditions in boreal ecosystems. The FP revealed a significant positive correlation with G+, G− bacteria, and the total bacterial population (Fig. 6) consistent with observations in earlier findings^12^ in temperate ecosystems. The increase in above ground biomass is known to have a strong positive connection with the below ground soil health indicators^12,25,29,66–68^. This was evident in the strong associations between shifts in the active microbial populations (Figs 4–6), and increase forage production (Figs 5 and 6), RS-P_available_ (Fig. 5, Table 8) observed following intercropping forage soybean with silage corn under field conditions in cool climate boreal ecosystem. Collectively, reduction in RS‒pH, increased RS-APase activity, RS-P_available,_ as well as increased in the active fungi, protozoan and bacterial population appears to be the most important determinants of forage production (Figs 4–7), when corn and soybeans are cultivated as intercrops under cool climatic conditions in boreal environment. Similar relationships have been reported between agronomic performance and soil health indicators in various IC production systems under different climatic conditions^12,25,29,66–68^. This is the first study demonstrating that similar relationships exist when silage corn is intercropped with soybeans (vine or upright varieties) and cultivated on podzols under cool climatic conditions in boreal ecosystem. Soil microbes are known to mineralize organic matter and other sources of plant nutrients located in the soil, thus making them available to the plant for uptake, growth and productivity^12^. This is consistent with the significant correlations observed between RS-A_vailable_, and the active soil microbial population (Table 8). Similarly, IC can stimulate the enrichment of P solubilizing soil microbes or microbial species with enhanced soil phosphatase activities, thereby increasing the RS‒P_available_ during IC^12,29,68^. Thus, it appears, under cool climatic conditions in boreal environments, that the increased forage production in the IC production system was highly dependent on RS‒P_available_ and RS-APase activity, presumably through stimulation or modification of the active microbial community structure (Fig. 5, Tables 6–8). The enhanced microbial population observed in IC (Table 7) might be more efficient in mineralizing and mobilizing P in the root rhizosphere under cool climatic conditions in boreal ecosystem. This could be the mechanism through which the improved agronomic performance observed in the tested IC system may be related to the enhanced soil health status. Further experimentation at the molecular genetics and cellular levels are needed to confirm this mechanism and are the subject of future work in our research program.

## Conclusion

Consistent with the objective of this study. We observed that forage soybeans intercropped with silage corn resulted in significantly enhanced agronomic performance and forage production in cool climate boreal ecosystem. In general, corn intercropped with US genotypes displayed superior agronomic performance compared to when intercropped with VS.

Collectively, reduction in RS‒pH, and increased RS-APase activity, RS-P_available_, and the active fungi, protozoan and bacterial populations appear to be the most important determinants of the soil health status and improved forage production, when silage corn and forage soybeans are cultivated as intercrops in podzols under cool climatic conditions in boreal environment.

This study is the first to demonstrate that intercropping silage corn with forage soybeans is a suitable approach to increase forage production and enhanced the soil health status under cool climatic conditions in boreal ecosystems. This work will provide significant improvement in our knowledge to better understand agriculture production in boreal ecosystems or northern climates, particularly in the context of climate change and expanding global populations in these geographic regions where food security is anticipated to be a challenge in the future^1,2^.

## Methods

A two-year field research trial was conducted at Pynn’s Brook Agricultural Research Station, Pasadena, NL (49.0130°N, 57.5894°W), managed by the Department of Fisheries, and Land Resources, Government of Newfoundland and Labrador (NL), Canada. Two silage corn (C1: Yukon-R and C2: DKC26-28 RIB) obtained from Brett Young™ and Dekalb® respectively, and three forage soybean genotypes (S1: Big Fellow RR, S2: Game Keeper RR, (obtained from Delaware State University, USA), S3: Kester’s Bob White Trailing Soybean-vine type obtained from the (United States Department of Agriculture) were sown on June 20^th^ and May 30^th^ during 2016 and 2017 using a SAMCO seeding machine (SAMCO Agricultural Manufacturing, Limerick Ireland). The sowing of vine type soybean (S3) was carried out with a hand drill due to the small seed size with same line spacing (almost 1 m) as done with SAMCO system for other corn and soybean varieties. The silage corn genotypes were selected based on low corn heating units requirements^69^. There was a total of eleven treatments for the two corn and three soybean genotypes cultivated as either mono or intercrops (Table 1).

The following seeding rates were used for MC (corn: 77,100 seeds ha^−1^; soybean: 129,200 seeds ha^−1^) and IC (60% corn + 40% soybean; total 129,200 seeds ha^−1^) during both study years. Crop nutrient requirements were fulfilled through inorganic fertilizers using the regional recommended rates for MC or IC based on the soil nutrient status prior to planting (Supplementary Table S1).

Soybean seeds were inoculated with *Bradyrhizobium japonicum* @ 10 g kg^−1^ seeds^70^ before seeding. Herbicide application was carried out using roundup WeatherMax® (Monsanto Canada Inc) during both growing seasons for weed control. The crop was harvested on October 25^th^ and 13^th^ during 2016 and 2017, respectively.

The experiment was laid out in a randomized complete block design with three replications per treatment. Each experimental treatment plot was 5 m × 6 m in dimension. Weather data for both seasons were collected from a weather station located adjacent to the experimental plots and are reported in Fig. 1.

### Crop agronomic performance: Chlorophyll contents, final plant height, and forage production

The chlorophyll contents were measured using a portable chlorophyll meter (SPAD-502 Konica-Minolta, Japan) taken from the top three leaves of the corn and soybean plants at 65 and 77 days after sowing during both growing seasons. At the corn physiological maturity (R6), four plants in a transect were selected from the replicates of each experimental treatment, and the plant height measured. The same plants were then uprooted and separated into roots and shoot to measure the biomass production. Plant fresh weight was recorded, and a subsample was taken from each treatment to measure the dry matter percentage by drying in a forced air oven (Shel Lab®) at 65 °C for 72 h. Thereafter, the total forage production was calculated considering the dry matter percentage and total fresh biomass per treatment.

### Soil health status evaluation

To quantify the effects of IC and MC on the soil health status, soil samples were collected from the root rhizosphere to measure the active microbial community composition by analyzing the microbial membrane phospholipid fatty acids (PLFA), along with soil pH, soil available P and soil acid phosphatase activities (i.e. the rhizosphere active microbial community composition, soil available P, soil pH and acid phosphatase activity were used as indicators of the soil heath status in this study). At harvest, plants were uprooted gently, and soil samples near the roots were collected, additionally roots were shaken gently to collect all soil attached to the root surface to obtain the rhizosphere soil for further analysis. The rhizosphere soil was sieved through 2 mm meshes to remove plant roots, small stones, gravel etc. Aliquots of the fresh soil (4 g) was used for PLFA analysis, and the rest of the soil stored at −20 °C for testing RS-P_available_, RS-APase activity and soil pH.

### Rhizosphere soil pH

RS-pH was measured in a 1:2 (w/v) ratio soil solution in CaCl_2_ using a soil pH meter (METTLER TOLEDO, Canada)^71^. Briefly, 10 g of air-dried, sieved soil (2 mm) was weighed in 50 mL polypropylene centrifuge tubes, and 20 mL of 0.01 M CaCl_2_ was added. The soil solution was then mixed for 30 min on an orbital shaker (Innova^™^ 2300 Platform Shaker, New Brunswick Scientific, USA) at 120 rpm, then allowed to stand for 1 h before measuring the RS-pH.

### Rhizosphere soil acid phosphatase activity

RS-APase activity was measured using the modified methods of Tabatabai and Bremner^72^. Briefly, 1 g of 2 mm sieved soil was weighed and extracted in 1 mL of 0.09 M (pH 4.8) citrate buffer. Polypropylene centrifuge tubes containing soil and citrate buffer were then centrifuged (Heraeus™ Megafuge™ 16 Centrifuge Series) at 5000 rpm for 10 min. An aliquot (50 µL) of the supernatant was collected and RS-APase activity assessed after incubating for 30 min in the oven at 37 °C with 1 mM of 4-nitrophenyl phosphate (pNP) and 50 µL citrate buffer. The reaction was terminated immediately after incubation with 20 µL of 0.5 N sodium hydroxide (NaOH). The absorbance of the mixture was recorded at 405 nm using a spectrophotometer (BioTek™ Cytation™ 3 imaging reader, BioTek, VT USA.) and the RS-APase activity presented in μ mole pNP g^−1^ soil 30 min^−1^.

### Rhizosphere soil available phosphorus (RS-P_available_)

RS-P_available_ was analyzed using the Mehlich-3 extraction method^73^. Briefly, 2 g air dried soil was weighed in 50 mL Erlenmeyer flasks, and 20 mL of Mehlich-3 extractant solution (1:10 soil: extractant) was added. Flasks containing the mixture were shaken for 5 min on an orbital shaker at 120 rpm (Innova^™^ 2300 Platform Shaker, New Brunswick Scientific, USA), and the filtrate recovered following filtration using Whatman 42 filter papers (Sigma Aldrich, ON. Canada). Aliquots of the filtrate was then analyzed using an AA3 Continuous Flow Analytical System (AA3HR, SEAL Analytical USA) to measure the phosphate content, which was then converted to total phosphate in the soil sample based on soil weight as follows:

$$Melich3\,P\,(mg\,k{g}^{-1})=[P\,con.\,Melich3\,extrant\,(mg\,{L}^{-1})]\,\times [(\frac{0.002\,L\,extract\,vol.}{0.002\,kg\,soil})]$$

### Phospholipid fatty acid (PLFA) analysis to determine active microbial biomass

A modified version of the Folch method^74^ was used to extract the soil PLFAs. Briefly, the total soil microbial fatty acids were extracted using 4 g sieved (2 mm) soil with 10 mL of chloroform-methanol, 2:1 (v/v). The sample mixture was sonicated (Q700 Sonicator, Fisherbrand™ UK.) for 5 min using the following parameters: (Amplitude 50; Pulse on time: 5 seconds; and pulse off time: 10 sec). The samples were kept in an ice bath to cool the samples during sonication. The sample mixture was then incubated at room temperature for 24 h. After incubation, the supernatant was filtered (Whatman 42 filter paper, Sigma Aldrich, ON. Canada), then dried under a gentle stream of nitrogen in pre-weighted sample vials. The total lipids extracted were resuspended in 2 mL chloroform and fractionated with a Visiprep™ SPE Vacuum Manifold and Discovery® DSC-Si SPE columns (50 μm, 70 Å, 100 mg/1 mL) (Sigma-Aldrich, ON. Canada) into neutral lipids, glycolipids, and phospholipids using (2.5 mL) chloroform, (4 mL) acetone and (2.5 mL) methanol, respectively. The phospholipid fractions were re-dissolved into 500 µL of methyl tert-butyl ether (MTBE), and aliquots (100 µL) of the phospholipid fractions derivatized using 50 µL trimethyl sulfonium hydroxide (TMSH) in 2 mL GC vials^75^. The mixture in the vials were vortexed and incubated for 30 min at room temperature. After incubation, 10 µL of the internal standard methyl nonadecanoate (C19:0 @ 160 µg/mL) were added to the samples in the vials, and the samples analyzed via gas chromatography-mass spectrometry (GC-MS) and gas chromatography-flame ionization detection (GC-FID).

### GC-MS/FID analysis of soil microbial PLFAs

GC-MS/FID analysis was conducted on a Thermo Scientific Trace-1300 gas chromatography (GC) coupled to a Thermo Scientific TSQ 8000 Triple Quadrupole mass spectrometer (MS) and a flame ionization detector (FID). GC-MS was used for peak identification, while GC-FID was used for quantification. Methylated fatty acids were separated with a DB23 high resolution column (30 m × 0.25 mm × 0.2 μm; Agilent Technology, Mississauga, Canada) using helium as the carrier gas at a flow rate of 1 mL min^−1^. One (1 μL) of each sample was injected in split less mode using a Tri-plus auto-sampler. The oven temperature was programed as follows: the initial oven temperature of 50 °C was held for 1 min, then programmed to increase at 20 °C min^−1^ to 175 °C, held for 1 min at 175 °C, then increased at 4 °C min^−1^ to 230 °C, where it was held for 5 min. The methylated PLFAs were identified through retention times comparison and mass spectra obtained from commercial standards (NIST database) (Thermo Scientific, ON. Canada; Supelco 37 Component FAME Mix, and Bacterial Acid Methyl Ester (BAME) Mix obtained from, Sigma Aldrich, ON, Canada). Quantification of individual PLFAs was done using standard curves prepared from the standard mixtures, and values presented as nmol g^−1^ soil. A total of 37 PLFAs were identified (Table 2) and 27 of them used as biomarkers to assess different microbial groups living in the soil (active microbial community composition) at the time of sampling.

### Calculations and statistical analysis

The LER is the relative land area needed for MC to produce the same yield attained by IC^76^. LER was measured to evaluate the effect of IC verses MC^12,76,77^ as given in following Equations:

1 $${{\rm{L}}}_{{\rm{C}}}=\frac{{{\rm{Yield}}}_{{\rm{corn}}{\rm{IC}}}}{{{\rm{Yield}}}_{{\rm{corn}}{\rm{MC}}}}$$

2 $${{\rm{L}}}_{{\rm{S}}}=\frac{{{\rm{Yield}}}_{{\rm{soybean}}{\rm{IC}}}}{{{\rm{Yield}}}_{{\rm{soybean}}{\rm{MC}}}}$$

3 $${\rm{LER}}={{\rm{L}}}_{{\rm{C}}}+{{\rm{L}}}_{{\rm{S}}}$$

where, L_C_ and L_S_ are the partial LER for intercropped corn and soybean, respectively. When the LER value is greater than 1, it indicates that an advantage is gained from IC compared to monocrop cropping system in terms of the use of environmental resources for plant growth and yield (total production). When the LER is equal to 1, it means IC has no advantage over MC in the use of environmental resources; and when the LER is less than 1, it means MC use resources more efficiently than IC for plant growth and yield^12,49,77^.

All statistical analyses were conducted using XLSTATS (Addinsoft Inc, Paris, France) and Statistix-10 software programs (Analytical Software, FL, USA), while graphs were created with Sigma Plot 13.0 (Systat Software Inc., San Jose, CA). All measurements of chemical parameters (PLFAs, dry matter, plant height etc.) were made in quadruplet. Analysis of variance (ANOVA) was used to determine the effects of treatments on chemical parameters. Where treatment effects were significant, the means were compared using Fisher’s LSD test at α = 0.05. The effects of IC or MC treatments on plant agronomic performance (biomass production or forage yield, plant height, chlorophyll content etc.), and soil health status indicators (soil pH, RS-APase, activities et) were evaluated in this study. Redundancy analysis (RDA) and Pearson’s correlation coefficients (r) were used to test the linear relationships between FP, (RS-APase) activities, RS-AP, active microbial composition and soil pH.

## Supplementary information

Supplementary Info
