# Supplementary material for: The potential of corn-soybean intercropping to improve the soil health status and biomass production in cool climate boreal ecosystems
Source: Sci Rep. 2019 Sep 11;9:13148. doi: 10.1038/s41598-019-49558-3 (PMC6739473; doi:10.1038/s41598-019-49558-3)
Supplement: Supplementary file 1 — Supplementary Info [file 41598_2019_49558_MOESM1_ESM.docx]

**Supplementary Information**

***of***

**The potential of corn-soybean intercropping to improve the soil health status and biomass production in cool climate boreal ecosystems**

Muhammad Zaeem^1,^*, Muhammad Nadeem^1,2^, Thu Huong Pham^1^, Waqar Ashiq^1^, Waqas Ali^1^, Syed Shah Mohioudin Gilani^1^, Sathya Elavarthi^3^, Vanessa Kavanagh^4^, Mumtaz Cheema^1^, Lakshman Galagedara^1^, Raymond Thomas^1,*^

^1^School of Science and the Environment, Grenfell Campus, Memorial University of Newfoundland, Corner Brook, A2H 5G5, Canada

^2^Department of Environmental Sciences, COMSATS University of Islamabad, Vehari 61100, Pakistan

^3^Department of Agriculture and Natural Resources Delaware State University, 1200 N Dupont Hwy, Dover, DE 19901, USA

^4^Department of Fisheries, and Land Resources, Government of Newfoundland and Labrador, A0L 1K0, Canada

Corresponding authors: Muhammad Zaeem: [mzaeem@grenfell.mun.ca](mailto:mzaeem@grenfell.mun.ca); Raymond Thomas: [rthomas@grenfell.mun.ca](mailto:rthomas@grenfell.mun.ca)

**Table S1.** Soil properties at the beginning of the growing season just prior to planting.

| **Soil Property** | **2016** | **2017** | **Soil Property** | **2016** | **2017** |
| --- | --- | --- | --- | --- | --- |
| pH | 6.4 | 6.8 | Zn (mg kg^-1^) | 0.6 | 1.0 |
| Organic matter (%) | 3.0 | 3.4 | Mn (mg kg^-1^) | 18.0 | 10.0 |
| N (%) | 81.0 | 68.0 | S (mg kg^-1^) | 14.0 | 17.0 |
| P (mg kg^-1^) | 38.0 | 35.0 | Fe (mg kg^-1^) | 150.0 | 233.0 |
| K (mg kg^-1^) | 38.0 | 35.0 | Ca (mg kg^-1^) | 1256.0 | 1426.0 |
| Na (mg kg^-1^) | 7.0 | 5.0 | Mg (mg kg^-1^) | 265.0 | 322.0 |

**Table S2.** Land Equivalent Ratio (LER) for intercropping treatments during both growing seasons.

| **LER (2016-17)** | | | |
| --- | --- | --- | --- |
| **Treatments** | **C** | **S** | **C+S** |
| S1C1 | 1.19±0.07 | 0.32±0.03 | 1.50±0.07 |
| S2C1 | 1.19±0.03 | 0.34±0.04 | 1.53±0.01 |
| S3C1 | 1.16±0.07 | 0.42±0.03 | 1.58±0.04 |
| S1C2 | 1.22±0.03 | 0.27±0.03 | 1.49±0.04 |
| S2C2 | 1.26±0.12 | 0.32±0.06 | 1.58±0.12 |
| S3C2 | 1.21±0.02 | 0.34±0.02 | 1.56±0.04 |
| **Average** | 1.21±0.02 | 0.33±0.02 | 1.54±0.02 |
| **LSD _0.05_** | NS | NS | NS |

Values are means ± standard errors. LER: Land Equivalent Ratio; C: corn; S: soybean; and C+S: corn + soybean. S1C1: Big Fellow RR + Yukon-R; S2C1: Game Keeper RR + Yukon-R; S3C1: Kester’s Bob White Trailing Soybean + Yukon-R; S1C2: Big Fellow RR + DKC26-28RIB; S2C2: Game Keeper RR + DKC26-28RIB; S3C2: Kester’s Bob White Trailing Soybean + DKC26-28RIB.
